# Breeding Vegetables with Increased Content in Bioactive Phenolic Acids

**DOI:** 10.3390/molecules201018464

**Published:** 2015-10-09

**Authors:** Prashant Kaushik, Isabel Andújar, Santiago Vilanova, Mariola Plazas, Pietro Gramazio, Francisco Javier Herraiz, Navjot Singh Brar, Jaime Prohens

**Affiliations:** 1 Instituto de Conservación y Mejora de la Agrodiversidad Valenciana, Universitat Politècnica de València, Camino de Vera 14, Valencia 46022, Spain; E-Mails: prakau@doctor.upv.es (P.K.); isanpe@upvnet.upv.es (I.A.); sanvina@upvnet.upv.es (S.V.); maplaav@btc.upv.es (M.P.); piegra@upv.es (P.G.); fraherga @upvnet.upv.es (F.J.H.); 2 Department of Vegetable Science, Chaudhary Charan Singh Haryana Agricultural University, Hisar 125001, India; E-Mail: singh.navjotbrar@gmail.com

**Keywords:** breeding, bioactive properties, genetic variation, molecular markers, phenolic acids, vegetables

## Abstract

Vegetables represent a major source of phenolic acids, powerful antioxidants characterized by an organic carboxylic acid function and which present multiple properties beneficial for human health. In consequence, developing new varieties with enhanced content in phenolic acids is an increasingly important breeding objective. Major phenolic acids present in vegetables are derivatives of cinnamic acid and to a lesser extent of benzoic acid. A large diversity in phenolic acids content has been found among cultivars and wild relatives of many vegetable crops. Identification of sources of variation for phenolic acids content can be accomplished by screening germplasm collections, but also through morphological characteristics and origin, as well as by evaluating mutations in key genes. Gene action estimates together with relatively high values for heritability indicate that selection for enhanced phenolic acids content will be efficient. Modern genomics and biotechnological strategies, such as QTL detection, candidate genes approaches and genetic transformation, are powerful tools for identification of genomic regions and genes with a key role in accumulation of phenolic acids in vegetables. However, genetically increasing the content in phenolic acids may also affect other traits important for the success of a variety. We anticipate that the combination of conventional and modern strategies will facilitate the development of a new generation of vegetable varieties with enhanced content in phenolic acids.

## 1. Introduction

Plant breeding programs have mostly concentrated on yield improvement, resistance to diseases, tolerance to abiotic stresses, longer shelf life, early or late production, and varietal diversification. However, consumers are increasingly becoming aware of the potential benefits resulting from diets rich in fruits and vegetables for maintaining a good health and preventing diseases [1]. In this respect, the scientific literature provides a wealth of information that correlates a diet high in fruits and vegetables with better health and disease prevention [2,3]. This has stimulated a growing demand for vegetables with enhanced contents in bioactive compounds. Many bioactive molecules derived from vegetables are effective due to their antioxidant activity, which prevents the formation of reactive oxygen, nitrogen, hydroxyl and lipid species, by scavenging free radicals or by repairing or removing damaged molecules [4,5]. The most relevant antioxidant bioactive molecules found in fruits and vegetables generally include hydrosoluble vitamins, carotenoids, and phenolics [6,7,8]. Occasionally, other classes of molecules, like glucosinolates in the case of brassicas [9], have relevant bioactive properties that contribute to the functionality of fruits and vegetables.

Among the major groups of bioactive compounds of vegetables, phenolic acids (molecules containing a phenolic ring and an organic carboxylic acid function) are becoming the focus of attention of many researchers given their properties for human health and their relative abundance in vegetables (Table 1). Phenolic acids are one of the diverse classes of the many different phenolic compounds synthesized by plants and are commonly found in plant-derived foods [10,11,12]. The bioactive properties of phenolic acids from vegetables are numerous (see below in the section “Properties of phenolic acids”). This has resulted in an increasing interest in breeding for enhanced content in phenolic acids content in vegetables [13,14].

Increasing the content in phenolic acids content of vegetables can be achieved by a variety of means, including development of improved cultivars, use of specific cultivation conditions, and application of postharvest treatments [15]. In this review, we will focus on breeding new cultivars with improved content in phenolic acids. This will require identifying the phenolic acid compounds most important and abundant in vegetables, the search for sources of variation (including crop wild relatives) with potential as breeding materials, and discussion of breeding strategies and biotechnological approaches appropriated for developing new vegetable varieties with enhanced content in phenolic acids.

**Table 1:** Average contents of total phenolic acids in different vegetables (mg/100 g of fresh weight) ranked according their average concentration (adapted from [12]).

Vegetable	Total Phenolic Acids [mg/100 g fw]	Major Soluble Phenolic Acids
Eggplant (*Solanum melongena*)	32.0	chlorogenic
Carrot (*Daucus carota*)	29.5	chlorogenic, caffeic, protocatechuic
Red beet (*Beta vulgaris*)	27.0	ferulic
Basil (*Ocimum basilicum*)	22.0	chlorogenic
Broccoli (*Brassica oleracea var. italica*)	15.0	sinapic, caffeic
Radish (*Raphanus sativus var. sativus*)	12.0	*p*-coumaric, ferulic
Spinach (*Spinacia oleracea*)	11.0	chlorogenic, protocatechuic, gallic
Chinese cabbage (*Brassica pekinensis*)	7.7	sinapic, chlorogenic
Parsley (*Petroselinum crispum*)	6.2	protocatechuic
Parsnip (*Pastinaca sativa*)	5.7	chlorogenic
Lettuce (*Lactuca sativa* var. *capitata*)	5.1	chlorogenic
Pepper (*Capsicum annuum*)	4.7	chlorogenic, *p*-coumaric, ferulic, protocatechuic
Cauliflower (*Brassica oleracea var. botrytis*)	4.6	*p-*coumaric, sinapic, chlorogenic
Turnip (*Brassica rapa*)	4.6	sinapic, ferulic, chlorogenic
White cabbage (*Brassica oleracea var. capitata* f. *alba*)	3.8	sinapic, *p*-coumaric
Grean bean (*Phaseolus vulgaris*)	3.5	chlorogenic, protocatechuic
Tomato (*Solanum esculentum*)	3.5	chlorogenic
Pea (*Pisum sativum*)	1.3	sinapic
Onion (*Allium cepa*)	1.0	protocatechuic, *p*-coumaric
Zucchini (*Cucurbita pepo*)	0.9	*p*-coumaric, caffeic
Cucumber (*Cucumis sativus*)	0.1	*p*-coumaric, ferulic

## 2. What Are Phenolic Acids?

**Figure 1:**
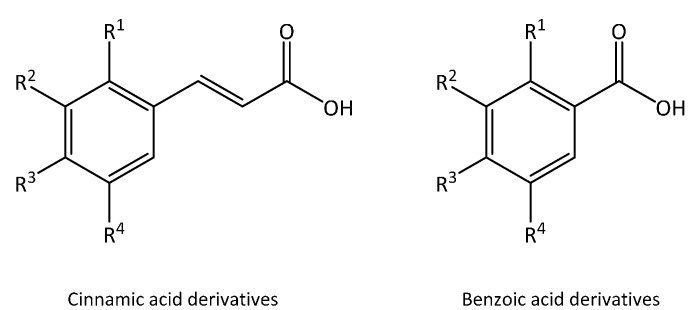
Chemical structures of major cinnamic and benzoic acids derivatives found in vegetables.

**Figure 2:**
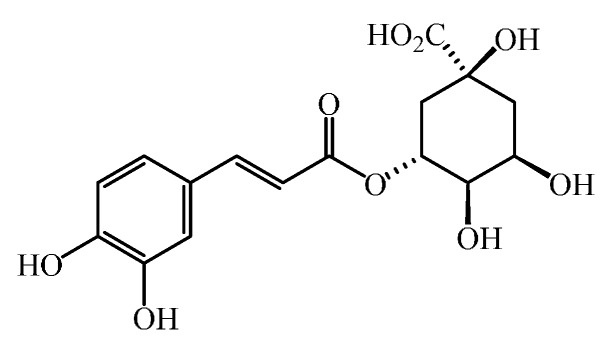
Structure of chlorogenic acid, a pre-eminent phenolic acid derivative present in many vegetables.

Phenolic acids are secondary metabolites characterized by the presence of an aromatic ring with an organic carboxylic acid functionality. Phenolic acids derive from benzoic and cinnamic acids; and although their basic structure remains the same, the number of the hydroxyl groups and their positions on the aromatic ring vary greatly resulting in different phenolic acids [16,17,18]. The most commonly found phenolic acids derived from benzoic acid in vegetables include gallic, *p*-hydroxybenzoic, syringic and vanillic acids, while those derived from cinnamic acid include caffeic, chlorogenic, ferulic, *p*-coumaric and sinapic acids [18] (Figure 1). Generally, the concentration of the derivatives of cinnamic acid in fruits and vegetables is higher than that of benzoic acid, except for certain red fruits and other plant products [19]. In this respect, chlorogenic acid, which is caffeic acid esterified with quinic acid (Figure 2), is pre-eminent among phenolic acids in many vegetables [12]. Phenolic acids can be found in plant tissues either as in a free or, more frequently, in a bound form. The bound fraction is generally found as esters, glycosides or in complexes [20,21].

**Table:** 

Substitution	Cinnamic Acid Derivatives	Benzoic Acid Derivatives
R^3^ = OH	*p-*Coumaric acid	*p-*Hydroxybenzoic acid
R^3^ = R^4^ = OH	Caffeic acid	Protocatechuic acid
R^2^ = OCH_3_, R^3^ = OH	Ferulic acid	Vanillic acid
R^2^ = R^3^ = R^4^ = OH		Gallic acid
R^2^ = R^4^ = OCH_3_, R^3^= OH	Sinapic acid	Syringic acid
R^2^ = R^3^ = OH [plus the carboxylic group being esterified with quinic acid]	5-*O-*caffeoylquinic acid	

Apart from their interest for human health, phenolic acids are very important for the quality of plant-based foods: they are substrates for enzymatic browning, and may affect flavour [22,23]. Furthermore, phenolic acids are signaling molecules involved in plant-microbe interactions [24].

**Figure 3:**
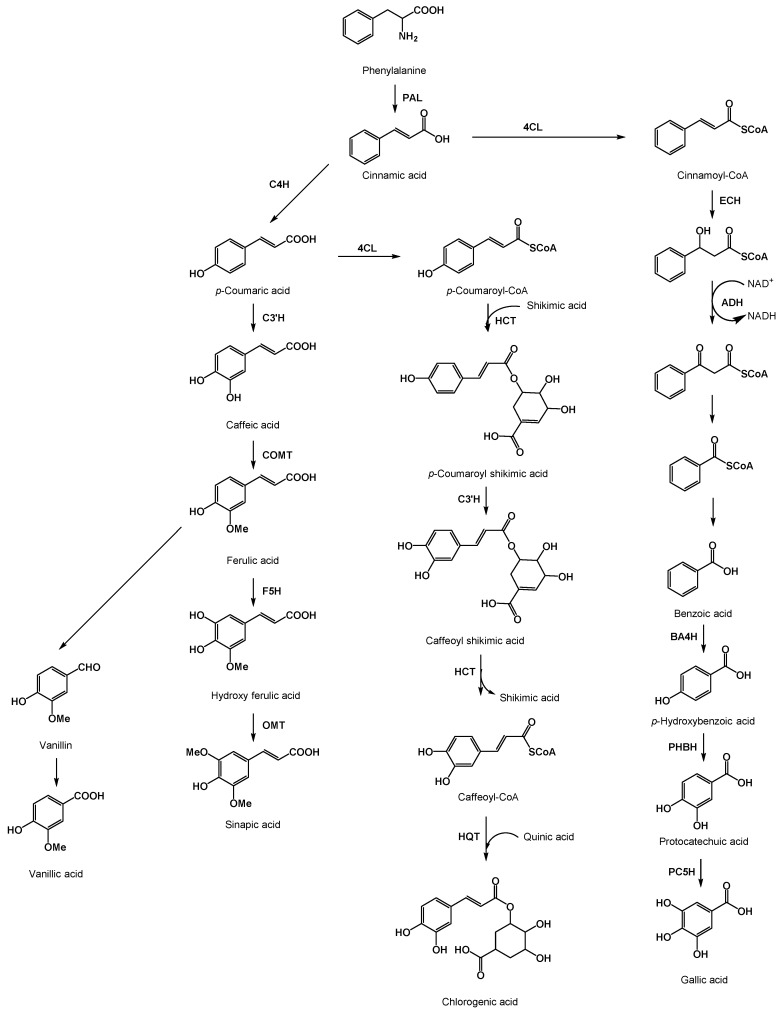
Schematic representation of some of the core biochemical pathways of major phenolic acids present in vegetables [25,29,30]. Enzymes involved in the pathways are indicated: PAL, phenylalanine ammonia lyase; C4H, cinnamate-4-hydroxylase; C3′H, p-coumarate 3′-hydroxylase; COMT, caffeic acid 3-*O*-methyltransferase; F5H, ferulate 5-hydroxylase; OMT, *O*-methyltransferase; 4CL 4-hydroxycinnamoyl-CoA-ligase; HCT, hydroxycinnamoyl-CoA shikimate/quinate hydroxycinnamoyl transferase; HQT, hydroxycinnamoyl-CoA quinate hydroxycinnamoyl transferase; ECH, enoyl-CoA hydratase; ADH, cinnamoyl alcohol dehydrogenase; BA4H, benzoic acid 4-hydroxylase; PHBH, p-hydroxybenzoic acid 3-hydroxylase; PC5H, protocatechuic acid 5-hydroxylase.

Knowledge of the biochemical pathway of phenolic acid is important for molecular breeding strategies. Phenolic acids are biosynthetically formed through the shikimic acid pathway from l-phenylalanine or, to a lesser extent, from l-tyrosine [25]. The core pathway for the biosynthesis of phenolic acids involves the synthesis of cinnamic acid from L-phenylalanine catalyzed by phenylalanine ammonia-lyase (PAL) [26]. Cinnamic acid is then further transformed, through the catalytic action of different enzymes (e.g., hydroxylases, methytransferases), into many varieties of phenolic acids, catalyzed by (Figure 3). Benzoic acid is synthesized from cinnamic acid via the β-oxidative pathway [27]. Regarding derivatives of benzoic acid, hydroxylation and methylation processes are similar to those occurring for cinnamic acid derivatives, resulting in derived phenolic acids (Figure 3) [25,28].

## 3. Bioactive Properties of Phenolic Acids

Phenolic acids are powerful antioxidants as they act by donating hydrogen or electrons, which can delay or inhibit the oxidation of bio-molecules (DNA, proteins, and lipids) [7]. The high correlation coefficient between phenolic acids content and antioxidant capacity in vegetables reveals that they play a main role in the bioactive properties of these plant products [31]. The antioxidant capacity of the phenolic acids depends on its structure, and it is higher in molecules with large number of hydroxyls [5]. In this respect, *in vitro* antioxidant activities of phenolic acids are even much higher than those of other major antioxidants present in vegetables, like vitamin C, E, and β-carotene [32].

There are many studies showing that phenolic acids are beneficial for human health and have a main role in preventing chronic diseases and therefore an adequate intake of phenolic acids should be part of a healthy and equilibrated diet [10,21,33,34]. Many epidemiological studies have revealed biological activities beneficial for human health of phenolic acids present in vegetables such as cardioprotective, anticarcinogenic, antimicrobial, hepatoprotective, antianxiety, antidiabetic and antiobesity properties [21,35,36,37,38,39].

## 4. Breeding for Increased Phenolic Acids Content

**Figure 4:**
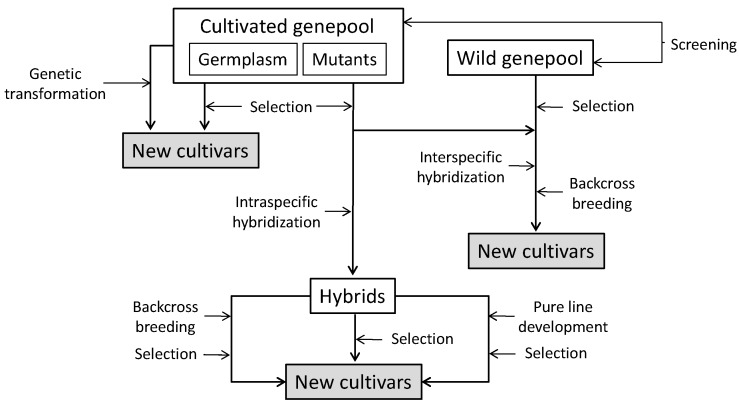
Summary of the main strategies for the development of new vegetable cultivars with increased content in phenolic acids. Screening and selection steps can be performed using phenotypic selection, marker assisted selection or both.

Conventional breeding techniques, based on selection and hybridization, have shown a high potential for enhancing the content of bioactive compounds in a wide range of plants [40,41]. Genetic improvement of phenolic acids content can be accomplished by different techniques, like simple mass selection or individual selection of plants with desirable characteristics for seed or vegetative propagation, or through the deliberate crossing of closely or distantly related individuals in order to produce new crop varieties or hybrids with increased contents (Figure 4). Genetic variation is necessary for efficient and successful selection and breeding for increased phenolic acid contents, and usually most of their variation is quantitative rather than qualitative [42,43]. Therefore, in general the conventional selection and breeding methods to be used for enhancing the content in phenolic acids in vegetables will be those of quantitative traits.

### 4.1. Identification of Sources of Variation

Large variation has been found for phenolics acid content among samples of cultivated species [43,44,45,46,47,48,49,50,51,52]. Table 2 presents the variation found in different vegetables for chlorogenic acid content, revealing that large differences may exist within a single species for a given phenolic acid. This variation, which can be of several fold differences among accessions of the same species, can be exploited to select varieties with higher content in phenolic acids or to identify parental materials for breeding programmes (Figure 4).

**Table 2:** Intraspecific variation for chlorogenic acid [g·kg^−1^ dw] content in different vegetables.

Vegetable	Chlorogenic Acid [g·kg^−1^]	References
Artichoke (*Cynara scolymus* L.)	0.4–7.3	[45]
Carrot (*Daucus carota*)	0.3–18.8	[46]
Chicory (*Cichorium intybus* L.)	0.1–0.9	[47]
Eggplant (*Solanum melongena*)	1.4–28.0	[48,49]
Lettuce (*Lactuca sativa* L.)	0.1–0.3	[50]
Pepper (*Capsicum annuum*)	0.7–0.9	[51]
Tomato (*Solanum esculentum*)	0.2–0.4	[52]

In some cases, morphological characteristics can provide an indication of the level of phenolic acids and therefore can be of interest for a preliminary selection of materials with potentially high content in phenolic acids. For example, Leja *et al.* [53] found that carrots with purple color roots possessed on average nine-fold higher phenolic acid content than carrots of other colors. Also, Vera-Guzmán *et al.* [54] reported that the color coordinates and chroma values presented a positive correlation with phenolic acid contents in Capsicum pepper. In the case of potato it was noticed that the pigmented cultivars like “Purple Majesty” and “Mountain Rose” contained considerably higher levels of chlorogenic acid isomers than the non-pigmented cultivars [55].

The origin may also be used on occasion for identification of sources of variation. For example, carrots of the Eastern (Asian) genepool often had higher content in phenolic acids than Western (European and American) genepool carrots [53]. Also, geographically-restricted Southeast Asian eggplants [*S. melongena* subsp. *ovigerum*] had a higher content in phenolic acids as well as greater diversity than eggplants from other regions [56].

Single mutations may represent an important source of variation for phenolic acid content (Figure 4). For example, mutants defective in light perception such as the high pigment [*hp*-1] mutant of tomato with increased fruit color result possess elevated chlorogenic acid content [57]. Also by utilizing somaclonal variation, a lettuce variety with high levels of chlorogenic acid was obtained [58].

Wild relatives are an important source of variability that can be used by plant breeders to develop vegetable varieties with increased contents in phenolic acids (Figure 4). For example, Meléndez-Martínez *et al.* [59] found that wild tomato species are a potential resource for increasing the phenolic acid content of tomato, as they presented higher concentrations than cultivated tomato. Also, in eggplant it has been found that artificial selection has resulted in a reduction in phenolic acids content and that wild relatives usually have higher contents in phenolic acids than cultivated eggplant [56]. In this crop, *S. incanum*, a wild relative of cultivated eggplant with high content in phenolic acids is being used in eggplant breeding programs as a source of variation for the introgression of this trait in the genetic background of eggplant by backcrossing [60,61]. Mennella *et al.* [62] studied the content in chlorogenic acid in lines of eggplant containing introgressions from three related species that had been selected for resistance to *Fusarium* and agronomic traits and found that *S. sodomaeum* introgression lines were highest in chlorogenic acid compared to introgression lines derived from two other species [*S. integrifolium* and *S. aethiopicum*]. Nonetheless, despite the interest of wild species as sources of variation for high content in phenolic acids, there are also associated disadvantages for breeding programmes, as they present many undesirable traits from the agronomic and commercial point of view [60,63,64]. As a result, selection against these traits has to be performed in the backcross generations. When traits to be removed are monogenic and dominant, selection will be much easier to be done than when are polygenic and with recessive inheritance.

### 4.2. Gene Action and Heritability

Knowledge of gene action and heritability values is important for devising efficient breeding strategies. However, there are few examples of determining these parameters for phenolic acids in vegetable crops. In a recent study by Prohens *et al.* [60], using a backcross population between cultivated eggplant and *S. incanum*, it was found that a simple additive-dominance model, in which only the additive variance was significant, explained the genetic variance for phenolic acid conjugate constituents. This indicates that genes from the wild species favoring the accumulation of phenolic acids should be in homozygosis in order to obtain higher contents in phenolic acids. Heritability studies for phenolic acid content of scarlet (*S. aethiopicum*) and gboma (*S. macrocarpon*) eggplants found moderate to high values of heritability for chlorogenic acid content and other phenolic acid contents and indicates that selection for these traits will be efficient in breeding programs [65].

The phenolic acid content is influenced by the growing environment and its interaction with the genotype [15,66,67]. For example, a recent study carried out by Stommel *et al.* [43] in order to evaluate the influence of the environment on fruit phenolics content in 12 different eggplant genotypes found a high genotype × environment interaction for phenolic acids content. However, these authors suggested that selection for stability could result in the selection of varieties with a reduced variability in phenolic acids content resulting from cultivation in different environments.

### 4.3. QTL and Candidate Genes for Phenolic Acids Content

New developments in molecular biology, genomics and metabolomics have provided new relevant information on the synthesis of phenolic acids. Detection and mapping of quantitative trait loci (QTL) in segregating populations or germplasm collections provides information of high interest for marker assisted selection and breeding [68]. Therefore, mapping major QTL for phenolic acids content will facilitate incorporation of this trait into élite vegetable cultivars through marker assisted selection.

Also, the candidate gene approach, which may be linked to the detection of QTLs, shows promise given that the genes involved in the phenolic acid synthesis pathway are known [Figure 3]. These genes are candidates for having a role in the accumulation of phenolic acids. In this respect, the genes codifying for enzymes involved in the core chlorogenic acid synthesis pathway in eggplant [PAL, C4H, 4CL, HCT, C3′H, HQT] were mapped on the eggplant genetic map, and it was shown that all of them, except for 4CL and HCT, were not linked, which may facilitate pyramiding of favorable alleles in a single variety [61]. The role of genes involved in the pathway of synthesis of phenolic acids on the accumulation of these compounds has been confirmed in some studies. For example, in tomato, the overexpression of the HQT gene increased the content in chlorogenic acid [30], while in potato it was found that the suppression of the expression of the HQT gene resulted in a reduction in the chlorogenic acid content of over 90% [69]. Other genes are also of interest for increasing the content in phenolic acids in vegetables. For example, in the case of tomato a major candidate gene associated to higher phenolic acid content expressing in fruit was identified as ERF1, which is a key gene in orchestrating the genes for phenolic content production in tomato [70]. In addition, the availability in Arabidopsis and other model plants of a large number of mutants of genes from the various branches of the phenylpropanoid pathway [71] may facilitate the identification in vegetables of candidate genes for increasing the content in phenolic acids.

## 5. Genetic Transformation for Increasing Phenolic Acids Content

Many transgenic strategies are available to enhance the nutritional value of crops; these strategies offer a rapid way to introduce desirable traits into elite verities [72], including the development of new cultivars with increased contents in phenolics (Figure 4). However, only a few studies in vegetables have been reported to increase phenolic acids content by using genetic transformation approach. For example, chlorogenic acid was increased up to 1.8-fold in tomato via constitutive expression of the hydroxycinnamoyltransferase HQT gene [30]. In an another recent study by Amaya *et al.* [73], the ectopic expression of the D-galacturonate reductase (FaGalUR) gene from strawberry aimed at increasing the ascorbic acid content led to a moderate increase in this antioxidant, but it simultaneously resulted in an increase of more than two-fold in chlorogenic acid content of tomato fruit. Also, the MYB family transcription factor AtMYB11 from *Arabidopsis* was noticed to be involved in the regulation of caffeoylquinic acid synthesis in tomato, as after transformation the transgenic plants had a significant increase in chlorogenic acid (18.1-fold) content compared to the non-transformed wild-type; also the contents of dicaffeoylquinic acids and tricaffeoylquinic acids were 68.0-fold and 108.4-fold higher in transgenic plants as compared to the wild-type. In the case of potato, constitutively expressed anti-sense strawberry chalcone synthase gene (CHS) resulted in a dramatic reduction of anthocyanin, flavonol and proanthocyanidines levels, while the phenylpropanoid pathway was upregulated leading to an increase in chlorogenic and caffeic acids contents [74].

Despite the potential of genetic transformation for increasing the content in phenolic acids in vegetables, the public acceptance of these genetically engineered crops is generally low [75]. In this respect, cisgenesis is a promising alternative to transgenesis for genetic engineering, with potentially less social rejection. Cisgenesis consists in the genetic transformation of a variety using only genetic material from the sexually compatible genepool [76]. In that case, it requires the identification of genes for phenolic acids from the sexually compatible genepool for introduction via genetic transformation.

## 6. Collateral Effects of Breeding for Phenolic Acids in Vegetables

Phenolic acids have relevant roles in plant life, including the response against biotic and abiotic stresses [77]. Apart from their bioactive properties for humans, phenolic acids have been associated with sensorial qualities of foods [78]. Additionally, the food industry has investigated the effects of phenolic acids on fruit maturation, enzymatic browning, and their roles as food preservatives [60,77]. In consequence, increasing phenolic acids content in vegetables may have an impact in other traits of interest, like tolerance to biotic and abiotic stress, browning, or flavor that should be taken into account in breeding new vegetable crops varieties.

### 6.1. Biotic and Abiotic Stresses

Phenolic acids are known to confer resistance to infection by a large number of pathogens, including fungi, bacteria, and viruses [79,80]. Increased synthesis of phenolic acids, which are incorporated to the cell wall of plants, takes place in response to biotic stress [81]. Phenolic acids are also known for their role in resistance to insect pests [82]. In this respect, resistance to thrips in chrysanthemum is attributed to higher chlorogenic and feruloyl quinic acid content [83]. Shivashankar *et al.* [84] found that resistance in chayote fruit against melon fly (*Bactrocera cucurbitae*) infestation was correlated with higher levels of *p*-coumaric acid. Nematoxic effects have also been reported for some acids like chlorogenic acid after nematode penetration [85]. It has also been demonstrated that phenolic acids may increase the tolerance to abiotic stresses. For example, salinity tolerance in lettuce is positively correlated with higher levels of chlorogenic acid [86]. In summary, the increase in the content in phenolic acids in vegetables may have a positive effect on resistance or tolerance to biotic and abiotic stresses. In this way, breeding for high content in bioactive phenolics in vegetables may lead to varieties more tolerant to stresses, which is an important objective in vegetable crops breeding.

### 6.2. Browning

Raising the total phenolic acids content may cause a negative effect on apparent quality of the fruit. In the case of vegetables when the tissue of interest is cut, phenolic acids, mostly stored in vacuoles, are oxidized resulting in brown coloration, *i.e*., enzymatic browning [22,87]. Enzymatic browning is mostly mediated by polyphenoloxidase enzymes. These oxidoreductases catalyze the hydroxylation of monophenols to diphenols. This reaction is comparatively slow and results in colorless products. Subsequently the same polyphenoloxidase enzymes catalyze the oxidation of diphenols to quinones, which is a fast reaction that yields brown colored products [88]. In consequence, a drawback of increasing the concentration of phenolic acids is that it may lead to a reduction in the apparent quality caused by the browning after exposure to the air [89]. However, it has been proposed that simultaneous selection for high content in phenolic acids combined with low activity PPO may result in a reduced or negligible impact on browning because of increased levels of phenolic acids in vegetables susceptible to enzymatic browning [61,90]. In this respect, it has been demonstrated, using transgenic approaches, that suppression of PPO activity results in a dramatic reduction of browning [91,92].

### 6.3. Flavour

Phenolic acids can contribute to the astringency and a have potential for causing bitterness in foods [93]. However, it has been found that phenolic acids, like chlorogenic acid, at the concentrations normally present in vegetables do not cause appreciable amount of bitterness [94], which is normally caused by other compounds like saponins, isocoumarins, glucosinolates and other compounds, like calcium, that may enhance bitterness [95,96,97]. In some cases, like in carrot, the content of isocoumarins increases with stress and can be responsible for the occasional bitter taste of carrots [96]. In the case, of phenolic acids, the cultivation environment may also have an important role in the phenolic acid levels [43], but it is unknown if this may have an effect on flavour of vegetables. Since literature is scarce on the effect of phenolic acids on flavour of vegetables further studies are needed to confirm the role of increased concentration of these acids on different flavour aspects of vegetables.

## 7. Future Prospects and Challenges

The development of vegetable crops with enhanced content in phenolic acids will benefit from the integration of conventional and modern techniques. In this respect, the germplasm collections of vegetable crops are largely unexplored regarding the content in phenolic acids and may allow the discovery of materials with high contents in phenolic acids [46,65,90]. Knowledge of candidate genes involved in the synthesis of phenolic acids [29,30,61] may also lead to the detection of new alleles in germplasm collections using EcoTILLING or sequencing techniques [98]. Also, the sequencing of genomes and the use of synteny among related species may be of great interest for the detection of genes and QTLs involved in phenolic acids accumulation in vegetable crops with limited genomic information [99]. Genome editing is also creating new opportunities for designing new varieties with increased content in phenolics through a non-transgenic approach [100,101]. With all the information already available and new developments, breeders have the challenge to develop a new generation of vegetables with enhanced bioactive properties resulting from an increased content in phenolic acids. These new varieties will have to be adapted to market requirements in terms of yield, shape, and organoleptic properties, which requires an integral breeding approach.
